# Neck lymph node status on survival of regionally recurrent or persistent nasopharyngeal carcinoma

**DOI:** 10.1038/s41598-020-62625-4

**Published:** 2020-03-27

**Authors:** David C. M. Yeung, Zenon Yeung, Eddy W. Y. Wong, Alexander C. Vlantis, Jason Y. K. Chan

**Affiliations:** 1 Department of Otorhinolaryngology, Head and Neck Surgery, The Chinese University of Hong Kong, Prince of Wales Hospital, Shatin, Hong Kong SAR China; 2 Department of Ear Nose & Throat, United Christian Hospital, Affiliated Teaching Unit of the Chinese University of Hong Kong, Kwun Tong, Hong Kong SAR China

**Keywords:** Surgical oncology, Prognostic markers

## Abstract

The aim of this study was to evaluate the impact of lymph node status from neck dissection pathological specimens on the survival for isolated regional nodal recurrence or persistence after primary treatment of nasopharyngeal carcinoma. Through a retrospective cohort study performed in an academic tertiary referral hospital in Hong Kong. Forty-six patients who underwent a salvage neck dissection between June 2001 and January 2013 for isolated regionally recurrent or persistent nasopharyngeal carcinoma was performed. Informed consent was waived for this retrospective study by The Joint CUHK-NTEC CREC. In the study forty-six patients had a salvage neck dissection for nodal failure with a mean age of 53 and 74% (34) were male. With a mean follow-up of 45.3 months, Overall survival, disease specific survival, loco-regional recurrence free survival, and regional recurrence free survival were 56.5%, 73.9%, 87.0%, and 91.3% respectively. For both univariate and multivariate analysis, patients with a number of positive lymph nodes more than 5 and a lymph node density more than 20% were significantly associated with poorer overall survival. Extracapsular spread and pathological cervical lymph node staging did not have an association with poorer survival. In conclusion, an absolute number of positive lymph nodes more than five and a lymph node density more than 20% were potentially useful prognostic factors affecting survival following a neck dissection for regional residual or recurrent nasopharyngeal carcinoma.

## Introduction

### Background

Nasopharyngeal carcinoma (NPC) has a high propensity for nodal metastasis with 49–85% of patients having lymph node metastases at presentation^1–3^. In addition, advanced nodal staging with N2 or above is associated with poorer overall survival, poorer disease free survival, and distant metastases^4^.

The nodal classification for NPC differed from that of other head and neck squamous cell carcinomas. Ho’s classification system in 1970^5^ was one of the first of its kind, shown to correlate with a poorer prognosis with an anatomically inferior level of cervical lymph node involvement. Ho’s staging system had been refined and incorporated to become its successor, the UICC AJCC staging system, with the 8^th^ edition which was published in the latest edition of the American Joint Committee on Cancer manual. The staging system for regional NPC status currently took into account the size, the laterality and the anatomical level or position of lymph node involvement. The pathological number of positive lymph nodes, total number of lymph nodes in specimen, and the density of lymph nodes were not incorporated.

The number of positive lymph nodes and lymph node density have been shown to be important prognostic factors in other non-NPC head and neck squamous cell carcinomas but there were no reports in regionally residual or recurrent NPC^5–8^.

## Objectives

This study aimed to evaluate the impact of the lymph node status on patient survival after surgery for regionally recurrent or persistent NPC and a possible practical threshold for risk stratification for further management.

## Materials and Methods

The study was approved by The Joint Chinese University of Hong Kong – New Territories East Cluster Clinical Research Ethics Committee (The Joint CUHK-NTEC CREC) and waived informed consent for the study. The study was performed in accordance with relevant guidelines and regulations.

### Study design

A retrospective review of all patients who underwent a salvage neck dissection for nodal recurrence or persistence after primary treatment for nasopharyngeal carcinoma, at an academic tertiary referral hospital in Hong Kong from June 2001 to January 2013 was performed.

### Data sources

Data was collected through the computer management system under Prince of Wale/s Hospital, Hospital Authority, Hong Kong. The data that was collected included demographics, clinical pathological characteristics, treatment, and follow-up status. All patients had a combination of ultrasound guided fine needle aspiration cytology(FNAC), computed tomography scan with contrast (CT), magnetic resonance imaging (MRI) or positron emission tomography/computed tomography

(PET-CT) pre-operatively to confirm status of regionally recurrent or persistent disease. All personal data involved was kept confidential during the review of cases for this retrospective study.

### Potential bias

There was potential selection bias as subjects were those who consented to neck dissection from a tertiary referral center covering half of Hong Kong, and may have over-represented the target population. Another potential source of bias is classification bias due to different methods of diagnosis were used in determining presence of regional recurrence.

### Study size and participants

There were 46 participants in this study. The number was a accumulation of patients from June 2001 to January 2013 in a tertiary referral center.

All patients underwent either a modified radical or a radical neck dissection. The extent of surgery depended on the extent of extra-nodal extension at presentation, namely, obvious invasion to the skin, dermis, surrounding soft tissue, spinal accessory nerve involvement, internal jugular vein adhesion or invasion, or where extra-nodal extension was demonstrated on pre-operative imaging. Bilateral neck dissections were performed for clinically or radiologically suspicious disease. Cases of cytology or histologically confirmed nodal recurrence in both sides of the neck had underwent bilateral neck dissections. Patients who were undergoing palliative surgery or carrying a second primary malignancy were excluded. Patients who had pathological N0 disease were excluded from survival analysis. All cases were staged according to the American Joint Committee on Cancer TNM Staging 6th edition.

### Statistical methods

The total number of lymph nodes was defined as the sum of all lymph nodes from the patient, regardless of being pathologically positive or negative for malignancy. lymph node density was calculated by dividing the number of positive lymph nodes by the total number of lymph nodes in the neck dissection specimen, and converting the value into a percentage.

Univariate analysis of survival was performed using the Kaplan-Meier with log rank test for significance. Multivariate analysis was done with Cox Proportional hazards regression. Overall survival(OS) was defined as the time from the date of surgery to the date of death from any cause or last follow-up. Disease specific survival(DSS) was defined as the time from the date of surgery to the date of death from locoregional recurrence or distant metastasis. Loco-regional recurrence or relapse free survival(LRRFS) was calculated from the date of surgery to the date of either local or regional recurrence, and regional recurrence free survival(RRFS) being only for regional recurrence. The cut-off values used for total number of lymph nodes, number of positive lymph nodes and lymph node density were determined by constructing a receiver operator characteristic(ROC) curve, and using the value where sensitivity was closest to specificity. The level of significance for all analyses was taken as p < 0.05. Data were tabulated using Microsoft Excel and were subsequently analyzed using SPSS version 21.0 (SPSS, Chicago, IL).

### Compliance with ethical standards

The study participants’ identifying information were replaced with subject labels to ensure data confidentiality of sensitive data and anonymity. All the authors in the manuscript had agreed for authorship, had read and approved the manuscript, and had given consent for submission and subsequent publication of the manuscript.

### Ethical approval

This study was approved by The Joint Chinese University of Hong Kong – New Territories East Cluster Clinical Research Ethics Committee (The Joint CUHK-NTEC CREC). The study was performed in accordance with relevant guidelines and regulations.

### Informed consent

No consent was taken for this study being retrospective in nature and that the involved data was de-identified.

## Results

### Participants and descriptive data

Forty-six patients with regionally recurrent or persistent NPC underwent a salvage neck dissection at the Prince of Wales Hospital between June 2001 and January 2013 with demographics and baseline characteristics summarized in Table 1. There was a male predominance of 74% (34 patients). The mean age was 53 years old (range 31–90). Mean follow up time from neck dissection was 44.67 months (range 0–151). Seven patients were lost to follow up after the first year. There were no pathological N0 patients in the cohort and all were EBER positive and associated with EBV.

**Table 1:** Demographics.

Age mean, years (range)	53 (31–90)
**Gender**	
Male	34 (74%)
Female	12 (26%)
**Smoking history**	
Non-smoker	24
Ex-smoker	8
Smoker	10
Unknown	4
**Drinking history**	
Non-drinker	29
Drinker	12
Unknown	5
Follow-up duration, mean, months (range)	44.7 (0–151)
**Initial T Classification**	
T1	15
T2	17
T3	11
T4	3
**Initial N Classification**	
N0	2
N1	4
N2	19
N3a	6
N3b	5

This table is a summary of the demographics and baseline charactersitcs of all subjects.

For primary treatment, 19 patients had radiotherapy alone to their nasopharynx and neck, while 27 patients had cisplatin based concurrent chemo-irradiation. Forty-four patients (95.7%) had nodal disease at the time of their initial treatment for NPC.

For the diagnosis of regional recurrence, thirty-two patients (70%) were diagnosed by ultrasound, 19 (41%) had contrast CT, 19 had PET-CT (41%), and 23 had MRI (50%). The 14 patients (30%) who did not have ultrasound, the diagnosis was either by a contrast CT, MRI, PET-CT or a combination of those three. For those with radiologically or clinically suspicious lymph nodes, 37 patients (80%) had FNAC with 32 patients with positive results, which was 86% of those who had FNAC. The remainder of those with heavily suspicious lymph nodes despite an inconclusive or benign FNAC result had proceeded to neck dissection. Local recurrence was screened out with an endoscopic exam of the nasopharynx (9 patients, 20% of participants) alone or in addition to a contrast CT, MRI, or PET-CT in the remaining.

Thirty-six patients had a unilateral neck dissection and ten patients had bilateral neck dissections, resulting in a total of 56 neck dissections performed in the study period. Specifics of neck dissection is summarized in Table 2. Of the neck dissections, 33 were on the left and 23 on the right. Also, 26 were radical neck dissections and 30 were modified radical neck dissections. On pathological examination of the neck dissection specimen, 57% had N1 disease, 39% had N2 disease and 4% had N3 disease.

**Table 2:** Neck dissection specifics.

Total sides of neck dissection	56
Unilateral neck dissection	36
Bilateral neck dissection	10
**Type of Neck dissection per side**	
Radical Neck Dissection	26 (46.4%)
Modified Radical Neck Dissection	30 (53.6%)
**Positive neck levels per side of neck dissection**	
I	11 (19.6%)
II	32 (57.1%)
III	18 (32.1%)
IV	10 (17.9%)
V	6 (10.7%)
VI	3 (5.3%)
**Pathological N Classification**	
N0	0
N1	26 (57%)
N2	18 (39%)
N3a	1 (2%)
N3b	1 (2%)
Extracapsular spread	31 (67.4%)
Number of lymph nodes examined, mean (range)	26.4 (1–90)
Lymph node density, mean, percent (range)	22.6% (1.82–100%)
Number of positive lymph nodes, mean (range)	4.3 (0–14)

This table summarizes the specifics of neck dissection of all subjects.

### Outcome data

Level II was the most commonly involved cervical nodal region with malignant lymph nodes identified at this level in 32 neck dissection specimens (57.1%), followed by level III with positive nodes in 18 specimens (32.1%), level I with positive nodes in 11 specimens (19.6%) and level IV with positive nodes in 10 specimens (17.9%). Level V and level VI (when it was dissected) were the least commonly involved levels with nodal involvement in six (10.7%) and three specimens (5.3%), respectively.

Extracapsular spread was present in 31 (67.4%) patients. The mean number of positive lymph nodes, total number of lymph node examined and lymph node densities were 4.3 (range 0–14), 26.4 (range 1–90) and 22.6% (range 1.82–100%) respectively. Patients with extracapsular spread were seen for discussion of adjuvant therapy.

A total of 23 patients (50%) had consented for adjuvant therapy. The types of adjuvant therapy given were radiotherapy (12 patients), brachytherapy (8 patients), brachytherapy with chemotherapy (1 patient), and chemotherapy alone (1 patient). 18 out of the 23 patients completed their adjuvant regimes. All 4 patients of the 23 who did not complete their adjuvant regimes had 100% 5-year overall-mortality, while 11 patients survived (70%) after 5 years. Those who survived with completed post-adjuvant therapy were 6 from the adjuvant therapy group (50% from adjuvant radiotherapy group) and 4 from the brachytherapy group (50% from brachytherapy group).

A total number of 12 patients (26.1%) had post-surgery locoregional recurrence. The mean time to locoregional recurrence after neck dissection was 21 months (6–56 months). Of the patients with locoregional recurrence after neck dissection, 3 patients (6.5%) had an isolated regional recurrence, 4 patients (8.7%) had an isolated local recurrence. 7 patients (15.2%) had both local and regional recurrence. Two patients (4.3%) had both an isolated regional recurrence and distant metastasis, and 4 patients (8.7%) had locoregional recurrence with distant metastasis. Positive number of lymph nodes, lymph node density, Extracapsular spread, and total number of lymph node from neck dissection specimen regardless of positive or negative showed no significant association with post-surgery locoregional recurrence.

When ROC curves were plotted for number of positive lymph nodes and lymph node density, and total number of lymph nodes in specimen, the cut-off values were determined to be 5 lymph nodes(sensitivity = 0.61, specificity = 0.68) and 20 percent(sensitivity = 0.72, specificity = 0.71), and 12 lymph nodes(sensitivity = 0.80, specificity = 0.96) respectively.

### Main results

**Figure 1:**
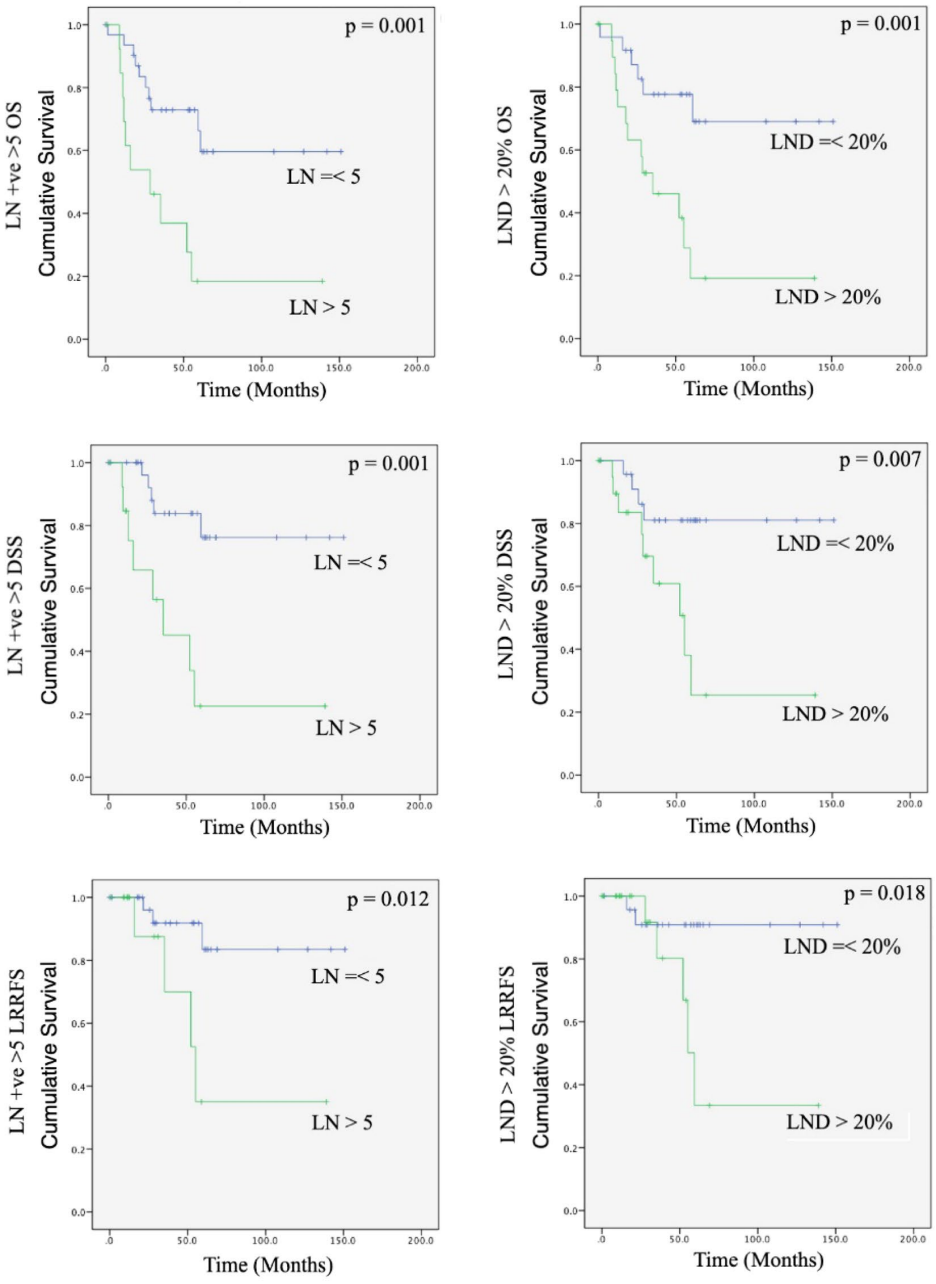
Kaplan Meier survival curves for number of positive lymph nodes more than five and lymph node density more than twenty percent. Notes: OS = overall survival; DSS = disease specific survival; LRRFS = locoregional recurrence free survival. LN + ve > 5: Number of positive lymph nodes more than five; LND > 20%: lymph node density more than twenty percent.

The OS, DSS, LRRFS, and RRFS for our cohort, was 56.50%, 73.90%, 87%, and 91.30% respectively. Kaplan-Meier survival analysis was constructed for lymph node analyses and baseline characteristics, and results were summarized in Table 3. The analysis showed that patients with higher number of positive lymph nodes had significantly worse overall survival, (p < 0.01), DSS (p < 0.01), and LRRFS (p < 0.01). Patients with a number of positive lymph nodes more than 5 had significantly poorer OS (p < 0.01), DSS(p < 0.01), and LRRFS (p = 0.12) as shown in Fig. 1. Patients with higher lymph node density had a lower OS (p < 0.01), and DSS (p = 0.023). Those with lymph node density more than 20% showed significantly poorer OS (p < 0.01), DSS (p < 0.01), and LRRFS (p = 0.018) as seen in Fig. 1. The total number of lymph nodes showed no association with survival. Patients with higher pathological recurrent N staging also had significantly poorer OS (p < 0.01) and DSS (p < 0.01). Extracapsular spread showed no significant findings in survival. Patients who drink had poorer OS (p < 0.01) and DSS (p < 0.01). Neither age, smoking, nor sex of the patients were shown to have a significant impact on univariate analysis. The results of univariate analysis were summarized in Table 3.

**Table 3:** Univariate analysis.

Factors	OS	DSS	LRRFS
	p value	p value	p value
Number of positive lymph nodes	0.001*	0*	0.002*
Number of positive lymph nodes > 5	0.001*	0.001*	0.012*
Lymph node density	0.004*	0.023*	0.057
Lymph node density > 20%	0.001*	0.007*	0.018*
Total number of lymph nodes > 12	0.117	0.46	0.072
Pathological N staging	0*	0*	0.14
Extracapsular spread	0.4	0.39	0.056
Sex	0.46	0.71	0.95
Age	0.11	0.53	n/a
Smoking	0.55	0.92	0.85
Alcohol	0.004*	0*	0.88

This is the output for univariate analysis performed with Kaplan Meier survival estimates.

Notes: OS = overall survival; DSS = disease specific survival; LRRFS = locoregional recurrence free survival. LRRFS was not calculated for age due to censoring of data.

Univariate analysis of total number of lymph nodes as a continuous variable was not performed as data was censored.

Multivariate survival analysis with proportional hazards regression was constructed for baseline characteristics and lymph node analyses, with a summary of results in Table 4. Total lymph node number was excluded due to having no association with survival in the univariate analysis. Results showed that patients with a number of positive lymph nodes more than five had decreased OS (Hazard ratio = 6.93, 95% CI = 1.56–30.70, p = 0.01), DSS (Hazard ratio = 7.11, 95% CI 1.51–33.47, p = 0.01) and LRRFS (Hazard ratio = 15.53, 95% CI 1.24–194.50, p = 0.03). Having a lymph node density more than 20% showed a decreased in OS (Hazard ratio = 7.60, 95% CI 1.13–51.10, p = 0.037). For the baseline factors, patients who were drinkers had poorer OS (Hazard ratio = 13.25, 95% CI 2.62–66.95, p < 0.01). Being a non-smoker was a protective factor (Hazard ratio = 0.07, 95% CI 0.01–0.82, p = 0.03). Patients with an age more than 50 had significantly poorer OS (Hazard ratio = 10.50, 95% CI 1.66–66.42, p = 0.01) and DSS (Hazard ratio = 7.73, 95% CI 1.36–44.03, p = 0.02). Having a completed course of adjuvant therapy, pathological neck lymph node staging, and sex were not significantly associated with survival in the multivariate analysis.

**Table 4:** Multivariate analysis.

Covariates	p value	95% CI	Hazard Ratio
OS			
Sex	0.635	0.25–9.49	1.55
Age (>50)	*0.013	1.66–66.42	10.50
Non-smoker	*0.034	0.007–0.82	0.07
Alcohol	*0.002	2.62–66.95	13.25
LN + ve (>5)	*0.011	1.56–30.67	6.93
LND (>20%)	*0.037	1.13–51.09	7.60
N staging (> = 2)	0.197	0.10–1.6	0.40
Completed Adj.	0.798	0.31–4.51	1.19
**DSS**			
Sex	0.635	0.24–10.14	1.57
Age (>50)	*0.021	1.36–44.03	7.73
Non-smoker	0.496	0.029–5.52	0.40
Alcohol	0.143	0.621–27.00	4.09
LN + ve (>5)	*0.013	1.511–33.47	7.11
LND (>20%)	0.477	0.246–20.44	2.22
N staging (> = 2)	0.655	0.250–9.07	1.51
Completed Adj.	0.584	0.342–6.72	1.52
**LRRFS**			
Sex	0.711	0.08–39.02	1.79
Age (>50)	0.129	0.52–164.54	9.27
Non-smoker	0.269	0.001–7.84	0.07
Alcohol	0.316	0.126–613.26	8.78
LN + ve (>5)	*0.033	1.24–194.50	15.53
LND (>20%)	0.518	0.06–319.65	4.18
N staging (> = 2)	0.557	0.05–226.62	3.49
Completed Adj.	0.292	0.03–2.96	0.28

These are the outputs for multivariate analysis with proportional hazards regression for OS, DSS, and LRRFS.

Notes: OS = overall survival; DSS = disease specific survival; LRRFS = locoregional recurrence free survival. LN + ve (>5): Number of positive lymph nodes more than five; LND (>20%): lymph node density more than twenty percent; N staging (> = 2), Pathological N classification with N more than or equals to two; Completed Adj.: Patients who had a completed course adjuvant therapy.

95% interval and Hazard Ratio was not calculated for smokers as it was the reference category for smoking status in this regression model.

## Discussion

NPC was endemic in southeast Asia and southern China, with an incidence of 30–50 per 100,000 persons^9^, in contrast to the US and Europe where there are fewer than 1 case per 100,000 persons^10^. Due to the high rates of regional lymph node metastasis, the neck was empirically irradiated despite absence of clinical regional disease^11^. With that, re-irradiation for regional recurrence was unfavorable due to the association with high morbidity. Surgery instead was held to be the standard of treatment of regionally recurrent NPC.

For monitoring of regional recurrence after primary treatment for NPC, it was described CT and MRI have only limited value in detection of microscopic metastasis^12^ and they were poor in sensitivity for regional recurrence staging and extracapsular spread. This, however, could be supplemented by a PET-CT, which showed significant increase in uptake for disease with extracapsular spread^13^. PET-CT^12^ or recently whole body MRI^14^ have been shown to have superior sensitivities (87.3–95% and 90.9%, respectively) compared to CT (76%) or conventional MRI (78%) alone for detecting recurrences in nasopharyngeal carcinoma. A combined interpretation of a whole body MRI and PET-CT increases the sensitivity for detecting recurrent/residual tumor to 94.5%^14^. PET-CT or whole body MRI is the current practical investigation of choice to look for second recurrences, and as up to 22.9% of our patients eventually developed a distant metastasis. With regards to Ultrasound guided FNAC as confirmation of regional recurrence, previous studies have showed a low sensitivity to detect recurrent or persistent nodal disease in nasopharyngeal carcinoma^15–17^. The sensitivity was only 25–70% in a previously irradiated neck due to fibrosis of the lymph nodes and necrotic tissue. FNAC had a high positive predictive value of 100% but a negative predictive value of 42.9%^14^.

An elevated lymph node density has been shown to be a poor prognostic factor in head and neck cancers^7,18^, particularly for oral cavity squamous cell carcinoma^6,8,19^, laryngeal carcinomas^20^, and hypopharyngeal carcinomas^21^. Lymph node status was investigated for survival in locoregional recurrent NPC^22^. In that study, univariate analysis on survival was significant for lymph node density, which the authors have called lymph node ratio, as well as the total number of pathologically examined lymph nodes. However, the multivariate analysis was insignificant for both lymph node density, total number of examined lymph nodes, or any of the lymph node status categorizations.

### Key findings

In this present study, all the patients had only regional recurrence on presentation without endoscopically or radiologically detected local recurrence. It was demonstrated in our cohort that OS was significantly associated with number of positive lymph nodes and lymph node density in both univariate and multivariate analysis. DSS was also significantly associated with number of positive lymph nodes in both the univariate and multivariate analysis. When the ROC determined thresholds were applied, survival decreased from 75.0% (24 out of 32 patients) to 28.6% (4 out of 14 patients) when the positive number of lymph nodes was more than 5. Similarly, survival decreased from 80.8% (19 out of 25 patients) to 38.1% (7 out of 21 patients) when lymph node density was higher than 20%. This showed that lymph node status prediction seemed more applicable to estimate survival in regional recurrence than for locoregional recurrence. Effects and results of this needed to be validated in other centers and localities.

Given the mean time to second recurrence was 21 months and the considerable number of patients who develop distant metastases, follow-up imaging could be considered at 1-year post salvage neck dissection to evaluate for disease recurrence.

For disease monitoring, recent investigations of serum and trans-oral nasopharyngeal brush biopsy for EBV-DNA without need of endoscopic guidance for detection of NPC showed promising results of have high sensitivity and specificity^23^. Its application may extend to monitoring of regional recurrence. Having an unfavorable serum EBV-DNA response at mid-course of RT was also shown to be an adverse prognosticator for treatment outcome^19^. Serum EBV DNA but may encounter difficulties in monitoring regional recurrences as the extensive fibrosis of the neck may impair the diffusion of the marker into the blood stream for detection, resulting in a low detection rate for loco-regional recurrence, ranging from ranged from 51 to 67%, while its detection rate for distant metastases was 86 to 96%^24,25^.

### Limitations

There were a few limitations in this study. Being a single center with only 46 patients, results might need to be validated by other centers for generalizability. The nature of being a retrospective cohort study also exposed this study to potential selection bias. The operations were done by several different surgeons with possible technical differences, resulting in different rates of disease recurrence.

Future research is required to look into the role of adjuvant systemic therapy for the high risk group of patients who have a lymph node density of >20% or who have a total number of positive lymph nodes of > 5. Recent studies in immunotherapy using autologous cytotoxic T lymphocytes targeting the Epstein-Barr virus^24–26^, or therapy targeted at the epidermal growth factor receptor (EGFR)^27^ show some promise and may prove useful in improving the outcome of these patients. Immune checkpoint ligand programmed death PD-1 or PD-L1 are also being actively studied. Activation of PD-1 or PD-L1 contributes to T-cell dysfunction and leads to tumorigenesis. Increased expression of PD-L1 has been demonstrated in EBV-associated nasopharyngeal carcinoma^28,29^ and immune checkpoint inhibitors may potentially be useful as an adjuvant treatment in nasopharyngeal carcinoma when further radiotherapy is not possible or considered harmful^30,31^.

## Conclusion

While the overall survival following a salvage neck dissection for regional NPC recurrence was 56.5% in this series, the regional recurrence free survival was 91.3%, which highlighted the role of this procedure in these patients. The absolute number of positive lymph nodes more than 5 and lymph node density more than 20% in the neck dissection specimen might be practical thresholds for risk stratification after salvage neck dissection for residual for recurrent regional NPC.
